# Elf autoencoder for unsupervised exploration of flat-band materials using electronic band structure fingerprints

**DOI:** 10.1038/s42005-025-01936-2

**Published:** 2025-01-17

**Authors:** Henry Kelbrick Pentz, Thomas Warford, Ivan Timokhin, Hongpeng Zhou, Qian Yang, Anupam Bhattacharya, Artem Mishchenko

**Affiliations:** 1 Department of Physics and Astronomy, the University of Manchester, Manchester, UK; 2 Department of Computer Science, the University of Manchester, Manchester, UK

**Keywords:** Electronic properties and materials, Electronic structure

## Abstract

Two-dimensional materials with flat electronic bands are promising for realising exotic quantum phenomena such as unconventional superconductivity and nontrivial topology. However, exploring their vast chemical space is a significant challenge. Here we introduce elf, an unsupervised convolutional autoencoder that encodes electronic band structure images into fingerprint vectors, enabling the autonomous clustering of materials by electronic properties beyond traditional chemical paradigms. Unsupervised visualisation of the fingerprint space then uncovers hidden chemical trends and identifies promising candidates based on similarities to well-studied exemplars. This approach complements high-throughput ab initio methods by rapidly screening candidates and guiding further investigations into the mechanisms underlying flat-band physics. The elf autoencoder is a powerful tool for autonomous discovery of unexplored flat-band materials, enabling unbiased identification of compounds with desirable electronic properties across the 2D chemical space.

## Abstract (web summary)

Machine-learning-based methods have become one of the key technologies for materials discovery. In this work, the authors introduce an unsupervised convolutional autoencoder that clusters materials by electronic band features rather than crystal structures and apply it to search for two-dimensional flat-band materials.

## Introduction

High-throughput computational methods based on machine learning are rapidly becoming the paradigm for next-generation materials discovery. These methods encompass the prediction of novel materials^1–8^ as well as automated synthesis^8–10^. However, testing the stability and properties of vast array of potential materials at the lab scale remains a significant bottleneck. Consequently, AI-based approaches to classify patterns among identified materials using their characteristic fingerprints (machine-learnable vector representations of material properties) are urgently needed. These techniques are crucial for understanding the emergent properties in computationally generated materials, such as flat band formation^11^, band topology, superconductivity, photovoltaic potential, catalytic behaviour, and more. Additionally, they enable predicted materials to be linked to compounds with experimentally confirmed properties. Using this approach, large sets of predicted materials can be analysed simultaneously, allowing candidates with the most promising properties to be efficiently flagged for further investigation.

Several previous studies have attempted to use unsupervised machine learning to explore the space of materials’ electronic band structures. For instance, using a selection of materials within a particular symmetry group and algorithmically exploring the feature space of energy eigenvalues along some of the high-symmetry lines in the reciprocal space, materials can be mapped onto t-distributed stochastic neighbour embedding (t-SNE) representation^12,13^. Alternatively, a parameter space of Hamiltonians can be navigated using an unsupervised path-finding algorithm, allowing to classify them topologically^14^. However, the supervised selection of feature space introduces bias, and fully unsupervised electronic band structure fingerprints will be beneficial for automating the discovery of materials with desirable electronic properties, such as flat bands.

Flat or nearly-flat bands, which are states with approximately the same energy, have attracted considerable attention for hosting exotic strongly correlated physics^15–19^. In two dimensions, ‘plane flat bands’ extend in both *k*_*x*_ and *k*_*y*_ directions, forming an extended planar manifold in reciprocal space. The suppression of kinetic energy in these bands facilitates strong electron-electron interactions, enabling phenomena such as chiral plasmons^20^, unconventional superconductivity, first observed in twisted bilayer graphene^21^, Chern insulator states^22^, and more.

This paper presents an approach to generate materials fingerprints based solely on electronic band structures from density functional theory (DFT) databases. Using a convolutional autoencoder (CAE), **elf**, we autonomously generate fingerprints to cluster materials by electronic band features without bias from crystal structure. The input to elf consists of band structure images automatically generated during preprocessing. Representing band structures as images has only minimal bias^11^ while offering several benefits. First, it leverages the strength of CAEs optimised for image processing. Second, it can analyse the vast corpus of research papers where band structures are presented as figures. Third, the visual format is intuitive for human researchers, enhancing AI interpretability. By employing unsupervised clustering algorithms, elf identifies fingerprints of 2D flat-band materials, uncovering novel chemical and electronic feature groups, thus extending the known flat-band paradigm. Two-dimensional embedded clustering plots map the chemistry of 2D flat-band materials, serving as a blueprint for high-throughput analysis of electronic and chemical properties. Scalable to any database containing band structure data, elf enables the autonomous exploration of computationally generated materials. Its robust encoding supports strong predictions of emergent electronic properties of grouped materials, particularly when accompanied by well-studied compounds in the same cluster, all at a very low computational cost. By detecting duplicates, elucidating chemical patterns, and clustering materials by electronic properties, elf provides a versatile alternative to structure-based fingerprints like CrystalNNFingerprint^23^. Applied here to 2D materials with flat bands identified in Ref. ^11^ using 2Dmatpedia^24^, one of the largest open 2D materials databases, this method complements high-throughput ab initio approaches, accelerating discovery across the vast 2D chemical space.

## Results

**Fig. 1:**
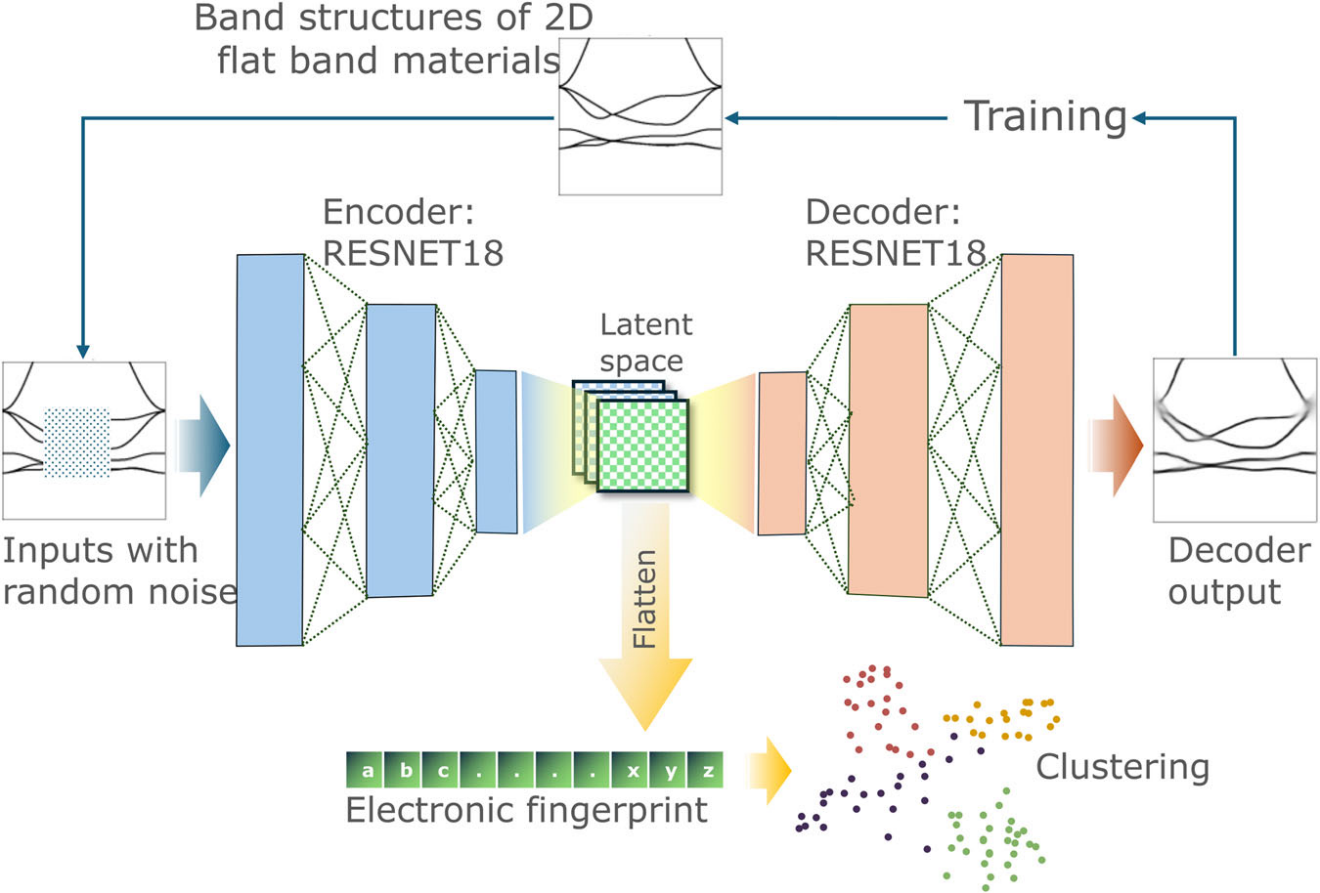
Elf network and fingerprint analysis pipeline for autoencoded band structure fingerprints. The convolutional autoencoder is trained to reproduce electronic band structure images within an energy range of  ±4 eV relative to the Fermi level. The process involves encoding these images into a compressed latent space representation. This compressed representation serves as the material fingerprint. The diagram outlines the steps of training the network, applying random noise to input images during training to enhance the learning of robust band structure features, and using the encoded representation for clustering materials based on their electronic properties. The reconstructed band structure of 2dm-1 (IrF_2_) is shown as an example, demonstrating the network’s ability to accurately capture and reproduce band structures even in the presence of noise.

Several attempts were recently reported to detect flat band materials in databases using computational screening and data mining techniques, creating repositories of well-documented 2D and 3D candidates^11,25–28^. Building on these efforts, we started this work from one such pool of 2127 flat band materials identified by Bhattacharya et al.^11^ using the 2Dmatpedia database^24^. For each of the flat-band materials, the band structure image data were encoded by a trained CAE elf, and the latent space representation was flattened to produce a 98-dimensional fingerprint vector. The training and subsequent fingerprint extraction process is shown in Fig. 1, with random Gaussian noise applied only during the training. We employed a ResNet18 architecture for both the encoder and decoder of elf, as its deep structure allows learning nuanced features of band structure images in comparison to shallow networks. Several ResNet models with different input image sizes and latent space dimensions were tested (further details in Supplementary Note 2). Optimum performance and accuracy for the chosen model were obtained with (224 × 224) input image size and (7 × 7 × 2 channels) latent space dimensions (further details in the Methods section ‘Network Training and Fingerprint’). Furthermore, by introducing random noise to the regions of input images during training, the network is forced to learn physically sensible connections between electronic state lines in the ‘noised’ regions, relying only on the shapes of the surrounding bands. This more challenging task helps to prevent overfitting by encouraging the learning of more general band structure features that are robust to small perturbations.

The set of fingerprints generated by the trained elf was then clustered using Hierarchical Density-Based Spatial Clustering of Applications with Noise (HDBSCAN)^29^. The optimal parameters for HDBSCAN were determined using the optimization procedure outlined in the Methods section ’Multi-stage clustering module’. Specifically, the minimum cluster size (*N*_*c*_) and the minimum sample size (*N*_*s*_) were set to 5 and 2, respectively. This clustering process identified 50 distinct clusters, while 1662 materials remained unclassified.

**Fig. 2:**
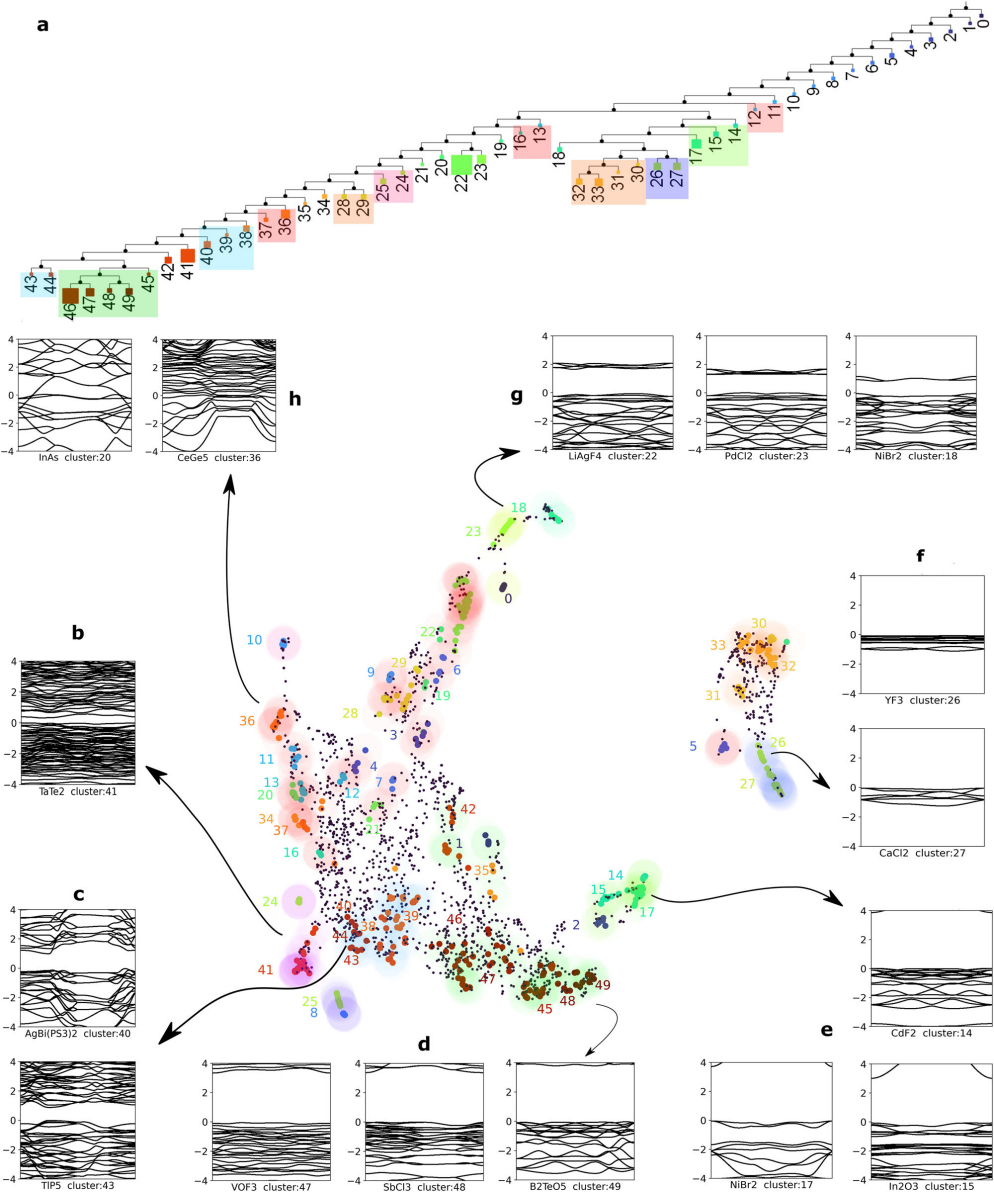
Visualization of the electronic fingerprint space and emergent chemical trends. Clustering using HDBSCAN and DBSCAN algorithms were combined with UMAP dimensionality reduction. **a** Phylogenetic condensed tree of the HDBSCAN clusters showing the relative sizes of the clusters. Clusters with similar defining features are grouped within the same coloured boxes. **b**–**h** Examples of band structures mapped onto UMAP plot for the machine-learned fingerprints, with Number of Neighbours (NN) = 10 and Minimum Distance (MD) = 0.1. The clusters determined by HDBSCAN are marked with an opaque colour, while the transparent shades from DBSCAN identify global trends and major types of band structures. The band structures of a few exemplars from a group of clusters are highlighted nearby to visualise their distinctive features: **b** Post-transition metal compounds and transition metal chalcogenides, with vanishing, indirect, or band overlap materials (potential semi-metallic phases). **c** 1-2 eV band gap materials with frequent crossings. **d** Wide band gap (>3 eV) semiconductors. **e** Insulators and large indirect band gap materials. **f** Metal oxides and halides. Insulators with strips of plane flat bands at the Fermi level. **g** Metal and transition metal halides with dispersive valence bands and plane-flat conduction bands. Within these groups, the band gap decreases from bottom (cluster 22) to top (cluster 18). **h** Flat bands with metallic band structures.

To visualise the distribution of materials in the machine-learned fingerprint space, we employed Uniform Manifold Approximation and Projection (UMAP)^30^. UMAP was applied with a Nearest Neighbour (NN) parameter of 10 and Minimum Distance (MD) parameter of 0.1, to reduce the high-dimensional fingerprint space to a 2D representation. Figure 2 presents the UMAP embedding, displaying the clustered materials in the reduced-dimensional space.

To further identify major groups of materials based on their electronic structure similarities, we applied an additional layer of clustering using the density-based (DBSCAN) algorithm^31^. This second clustering step was performed on the UMAP projected coordinates of the materials, excluding those that were left unclassified by HDBSCAN. The DBSCAN parameters were set to a maximum nearest neighbour distance (*ϵ*) on the UMAP projected plane of 0.8 and minimum cluster size (*S*_*min*_) of 5. This visualisation process created the shaded regions overlaid on the clusters in Fig. 2, highlighting the major classes of band structures present in the dataset.

During the density-based clustering process, HDBSCAN assigns a probability score to each data point. This score indicates the likelihood of the data point (material) belonging to its assigned cluster. This probability effectively measures the range of density cut-off values for which a given data point remains part of its assigned cluster throughout the clustering process. By sorting the materials within each cluster based on their membership probability, it is possible to identify ‘exemplar’ materials that are most representative of the properties characteristic of their respective clusters. These exemplar materials exhibit the highest membership probabilities within their clusters. A full list of clusters with the materials ordered by membership probability can be found in https://huggingface.co/datasets/2Dmatters/Elf_encoded_flat_band_materials/tree/main.

To visualise the separation and evolution of different band structure types across the UMAP embedding, we plotted band structure images corresponding to several exemplar materials from DBSCAN clusters alongside the UMAP chart, Fig. 2. The combination of the identified clusters and the UMAP embedding collectively form an electronic band structure genome for 2D flat-band materials, which serves as a comprehensive map of the diverse electronic properties found in these materials. This genome enables further exploration and a better understanding of the relationships among different flat-band materials based on their band structure characteristics. To further visualize the relationships among different types of band structures, we present a phylogenetic tree (Fig. 2a) that shows their hierarchical organisation. The hierarchy of the clusters is determined internally by the HDBSCAN algorithm, with additional details discussed in the Supplementary Note 3.

The leaves of the tree are opaque-coloured (same as in UMAP) squares, the size of which shows the relative size of the clusters. Adjacent leaves belonging to the same DBSCAN group are further grouped in the tree shown in transparent shades of the same colours. We can see that clusters 18, 30-31-32-33, 26-27 and 14-15-17, which form isolated groups on the right in UMAP, also form a separate branch in the tree. Clusters 45-46-47-48-49, 38-39-40, 36-37, 28-29, 13-16 and 24-25 also form adjacent leaves in the tree highlighting their similar origin.

### Analysis of global trends

The final stage of clustering using DBSCAN reveals distinct chemical patterns among the 2D flat-band materials. For example, clusters 26 and 27 predominantly contain halides, while clusters 24, 41, 25, and 8 are rich in transition metal chalcogenides. Other notable chemical trends are highlighted on the UMAP embedding in Fig. 2.

Interestingly, these chemical insights emerge solely from the unsupervised learning of electronic band structure features, without explicit input of the atomic composition. This can be attributed to the fact that materials containing elements from the same group in the periodic table often possess similar valence orbital structures, leading to comparable band arrangements and properties. Our convolutional autoencoder elf effectively captures these chemical patterns by learning the intrinsic similarities in the band structures.

**Fig. 3:**
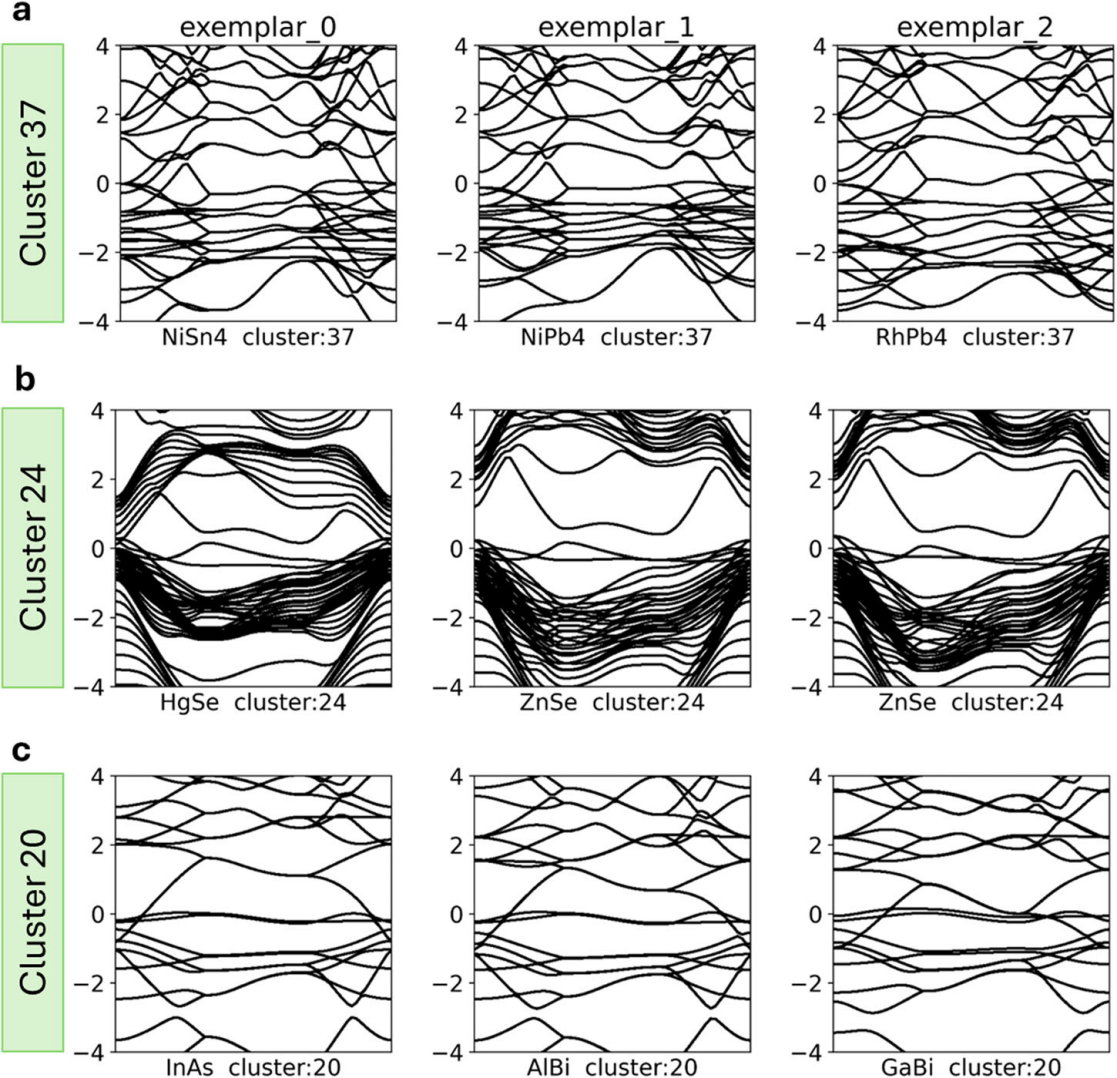
Homogeneity of band structure features within clusters. Band structures of the top three exemplar materials of clusters 37 (**a**), 24 (**b**), and 20 (**c**).

However, the clustering is not solely determined by the chemical composition. The precise features of the band structure, such as band gaps, crossings, and gradients, act as additional classification constraints. Consequently, each cluster contains materials with similar band structure characteristics, despite potential variations in their chemical composition or crystal structure. This is exemplified in clusters 38-39-40 (Fig. 3c, Dataset: https://huggingface.co/datasets/2Dmatters/Elf_encoded_flat_band_materials), where materials within each cluster exhibit characteristic band gaps, albeit deviations in the chemical composition. The convolutional nature of the autoencoder allows for a suitable margin of distortions and shifts in the electronic bands while still preserving the overall similarity. Materials with more pronounced deviations from the characteristic band structure of a cluster (exemplar band structure) are generally assigned lower membership probabilities.

The UMAP embedding in Fig. 2 reveals a global partitioning of the flat-band materials based on their electronic band structure features. Notably, metallic, semi-metallic, semiconducting, and insulating states are well separated in the latent space. This clear partitioning validates the effectiveness of the elf-based fingerprinting approach in capturing meaningful electronic features. Furthermore, several clusters (e.g., 47–49 in Fig. 2d and 23-22-18 in Fig. 2g) are characterized by the presence of dense, flat bands near the Fermi energy. These clusters are particularly interesting from a materials discovery perspective, as they may host stronger electron-electron correlations and potentially exhibit exotic phenomena. The identification of these sub-groups demonstrates the power of the unsupervised learning approach in uncovering materials with desirable electronic properties.

### Local trends

Within the individual clusters, HDBSCAN ensures a high degree of similarity among the band structure features of the clustered materials. Cluster 24, shown in Fig. 3b, is a prime example of this. The cluster consists primarily of semiconductors, with Hg and Cd-based materials exhibiting additional complexity in their band structures. Depending on their specific structure, these materials can exhibit semimetallic behaviour^32^, or a strain-tunable band gap, as observed in HgSe and HgTe, which can gradually transition into a topologically insulating phase^33^). The unique band features near the Fermi level in cluster 24 suggest that these materials could potentially lead to a range of useful (opto)electronic applications. Notably, most of the materials in this cluster were predicted to be stable as 2D monolayers, making them promising candidates for van der Waals heterostructures^34–36^.

One of the key advantages of our band structure-based fingerprinting approach is its ability to identify promising candidate materials that share electronic properties with well-studied compounds, even if their chemical compositions differ. By clustering materials based on their band structure similarity, we can flag computationally predicted compounds that have yet to be experimentally investigated but are likely to exhibit desirable properties.

For instance, cluster 46 contains 2dm-1072 Bi_2_O_3_, a wide-band-gap semiconductor frequently used in heterostructures for its optoelectronic properties^37,38^. Interestingly, the cluster also includes 2dm-3090 ZnMoO_4_ and 2dm-3226 Tl_2_SiSe_3_, which displays a nearly identical band gap and distribution of flat bands but has not been previously studied. Moreover, the properties of two-dimensional ZnMoO_4_ and Tl_2_SiSe_3_ have yet to be investigated in the literature. Based on its electronic similarity to Bi_2_O_3_, they are flagged as promising candidates for similar optoelectronic applications, warranting further investigation.

**Fig. 4:**
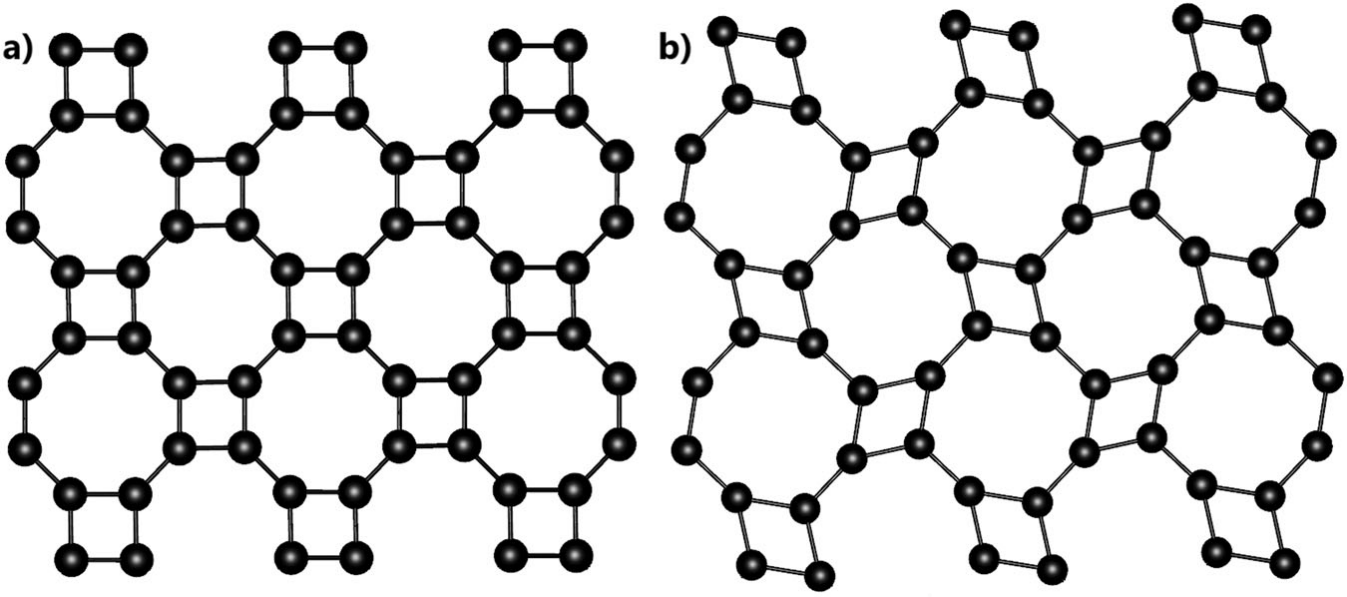
Schematics of square-octagonal lattice. **a** Symmetric square-octagon lattice structure. **b** Skewed square-octagon lattice structure^63^.

Another example of this predictive power is demonstrated by cluster 20. This cluster is anchored by the well-known semiconductor InAs (2dm-2474), which is considered one of the prime candidates for next-generation (opto)electronic devices due to its high mobility, large surface area, and direct band gap^39^. The cluster also contains several less-studied materials, such as AlBi (2dm-2252), a Rashba semiconductor^40^, and a group of thallium-based pnictides (2dm-2650 TlBi, 2dm-2847 TlSb and 2dm-2672 TlP, all of which display strikingly similar band structures to InAs (Fig. 3c). Notably, these materials share a square-octagonal lattice (Fig. 4), a structural motif known to host topological states^41,42^ and flat-band mediated correlated electron phenomena^43^. We predict that materials comprising cluster 20 will exhibit similarly promising electronic properties to InAs and are worthy of further investigation.

Interestingly, 2dm-2650 TlBi and 2dm-2847 TlSb in this cluster exhibit a unique asymmetrically skewed square-octagonal structure (Fig. 4b), a lattice that has not been previously reported to host flat bands. Despite this structural distortion, these materials remain close to the other symmetric square-octagon lattice materials in our fingerprint space. This underscores the ability of our approach to cluster materials with similar electronic properties even in the presence of structural variation.

This structural flexibility extends to the clustering of materials with entirely different crystal structures based on their common band features. For example, clusters 46-49 contain a variety of wide band gap (≈3 eV) flat-band semiconductors with different crystal structures. Notably, cluster 46 includes 2dm-3090 ZnMoO_4_, which exhibits a unique edge-sharing zigzag octahedra chain sublattice, a material whose flat-band formation mechanisms are yet to be understood.

While some of the observed flat-band clusters can be readily explained by well-known flat-band physics, such as the localisation of electron wavefunctions in the f-orbitals of lanthanides and actinides^27^, many others emerge beyond the known flat-band paradigm. For instance, there are bilayer flat-band structures commonly consisting of two stacked monolayers that individually exhibit flat bands, with well-studied examples including stacked square and Kagome arrangements. However, stacked centred-orthorhombic-square chains have only been reported very recently^11^.

By lowering the minimum cluster size to *N*_*c*_ = 4, we identify a cluster of four chemically similar materials: NdF_3_ (2dm-321), TbF_3_ (2dm-441), YF_3_ (2dm-553), and SmF_3_ (2dm-875). All of these compounds exhibit a bilayer sublattice structure composed of stacked centred-orthorhombic-square chains and possess flat electronic bands. The mechanisms leading to the emergence of flat bands in this type of lattice have yet to be uncovered.

Cluster 37, shown in Fig. 3a, contains a mixture of group 8-9 transition metals with group 14 elements (Si/Pb/Sn) with the *AB*_4_ and *AB*_3_ stoichiometries. The two groups of materials with these stoichiometries exhibit distinct tetragonal structures with different point groups (4/mm and 422, respectively). Further inspection of the orbital-projected band structures for these materials reveals that the nearly plane-flat bands arise from mixtures between the A and B elements, whose element groups are shared for both stoichiometries. This suggests that the plane-flat bands in this cluster likely result from a non-trivial interplay between the shared chemistry of these materials and the tetragonal *D*_4_ abstract symmetry group (shared by both 4/mm and 422 point groups) under which both lattices fall. More investigation is required to understand this interplay in detail, adding to the known non-trivial mechanisms that result in flat electronic bands beyond the traditional picture of orbital overlap within a single element sublattice^44^.

Clusters 26-27 are populated by alkaline metal halides which exhibit dense sets of particularly flat bands just below the Fermi level. These materials represent large band gap insulators.

### Comparison to structure fingerprints

While our auto-encoded fingerprint is highly effective at clustering materials based on their electronic properties, it is not completely robust to structural distortions. Small shifts in a lattice can alter the high-symmetry points in reciprocal space, which in turn affect the band structure image used as input to the network. However, the likelihood of this occurring in practice is minimal. We further inspected the clusters (see https://huggingface.co/datasets/2Dmatters/Elf_encoded_flat_band_materials/tree/main), and found that the ability to extend beyond structural similarity and cluster materials based on their emergent electronic features is a general capability of our approach.

**Fig. 5:**
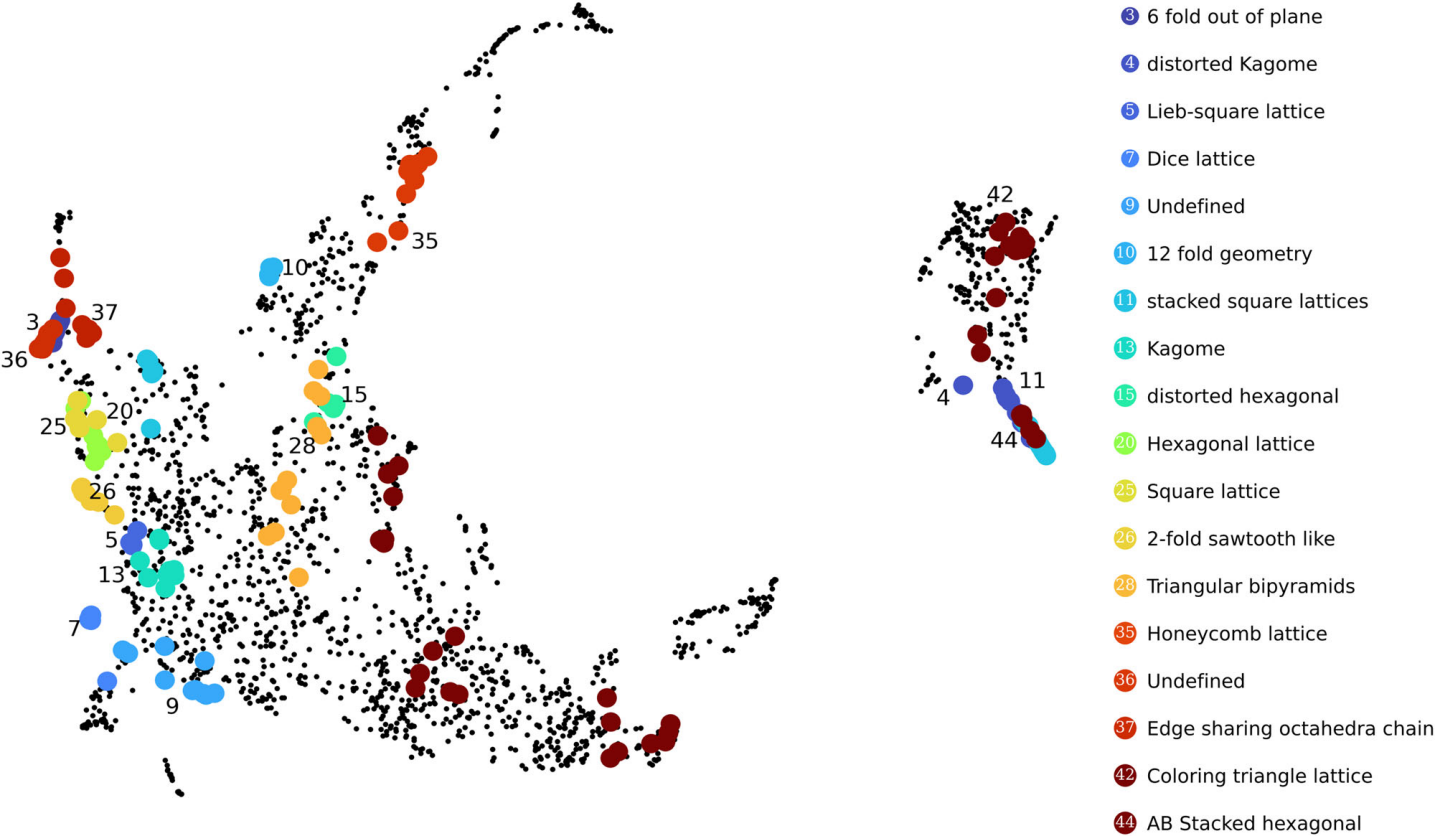
Clusters of iso-structural materials marked in the UMAP projected elf space. This plot helps to visualise the extent to which electronic fingerprints also cluster similar structures together. A structural fingerprint CNNF was used to find the similarity among structures. Out of 45 iso-structural groups, 18 also form clusters on elf UMAP space, showing the correlation between electronic and crystal structures.

To directly compare our elf fingerprints to structure-based fingerprints, we generated structural fingerprints for the flat band sublattices following our previous work^11^ for each of 2127 flat-band materials studied using CrystalNNFingerprint (CNNF)^23^. We then clustered these CNNF fingerprints using HDBSCAN, which resulted in a total of 45 clusters. To visualize the relationship between structural (CNNF) and electronic fingerprints (elf), we assigned each material in the elf UMAP space its corresponding CNNF cluster label. This allows us to identify any localized groupings of CNNF clusters within the electronic fingerprint space, thereby revealing correlations between structural and band-structure similarity. The resulting plot is shown in Fig. 5, with the identified structural motifs within each cluster listed on the right.

We find that out of the 45 CNNF clusters, 18 form localized groups of more than 5 materials within the electronic fingerprint space. This clearly demonstrates that certain structural motifs tend to give rise to similar band structures. For example, in Fig. 5, cluster 13 predominantly contains Kagome sublattices, cluster 7 hosts dice lattices, cluster 35 is composed of honeycomb lattices, and cluster 25 features square lattices. These lattice-specific groups form fairly isolated islands in the elf space.

This highlights the model’s capacity to differentiate non-trivial flat bands. In the absence of spin-orbit coupling, topological flat bands can be identified by a characteristic touching point with a dispersive band at a high-symmetry *k*-point. Additionally, topological flat bands arising from special lattices like Kagome or Lieb have a characteristic pattern in the band structure, which can also be identified in their tight-binding models^45^. Figure 5 illustrates that sublattices like Kagome and Dice form well-defined, low-spread clusters, confirming that these features are effectively learned and incorporated into our elf. Further examples include cluster 20 in Fig. 2, which contains materials with square-octagonal lattices known to support topological flat bands^46^, and cluster 16, which exhibits the Lieb-square lattice.

However, it is important to note that the number of CNNF clusters forming distinct groups in electronic fingerprint space is much lower than the total number of CNNF clusters. This implies that many materials sharing similar sublattice structures can indeed exhibit very different band structures. This can be attributed to the variations in the orbital composition of the materials, as well as differences in the atomic sizes and electronegativities of the constituent elements, which also play a role in determining the electronic distribution around the structural motifs.

### Duplicate detection

In general, when 2dmatpedia generates “bottom-up” materials via element substitution, the structure is allowed to relax to equilibrium bond lengths and angles without changing the crystal symmetries. Conversely, based on 2Dmatpedia’s “top-down” generation mechanism, we should expect that some layers exfoliated from unique 3D structures in the Materials Project will be equivalent in two dimensions. Subsequently, this could generate identical materials when the bottom-up element substitution chains intersect such that the constituent elements are also the same. These are duplicate materials, and most would have been removed by 2Dmatpedia using structure-matching tools available from the pymatgen library^47^.

Our algorithm enables the automatic detection of such duplicates, as demonstrated in Fig. 3b. In this example, we see two entries of ZnSe in cluster 24 exhibiting nearly identical band structures. Our elf detected several other pairs of duplicate materials with similar band structures, differing only by small structural distortions. The 2dmatpedia IDs of these materials are listed in Table 1, with each duplicate pair sharing the same chemical formula. Using this approach, we found up to 40 potential duplicate entries (listed in https://huggingface.co/datasets/2Dmatters/Elf_encoded_flat_band_materials/tree/main). However, further investigation is necessary to determine the stability of these materials within the accuracy of DFT calculations.

**Table 1:** Examples of the duplicates identified by elf

In_2_S	Tl_2_Te	PI_3_	Te_2_Se
2dm-1845	2dm-1554	2dm-495	2dm-1823
2dm-1986	2dm-1521	2dm-2009	2dm-1596

SrLaCl_5_	AsBr_3_	I_3_N	ZnSe
2dm-5260	2dm-2624	2dm-2010	2dm-2113
2dm-5422	2dm-4881	2dm-726	2dm-2321

We recommend verifying the comparative stabilities of the identified duplicate pairs and removing the less stable entries from the 2Dmatpedia database. Our fingerprint, based solely on electronic band structures, enables the identification of fundamentally equivalent materials that differ only by small structural distortions, setting it apart from structure-based methods. This capability is particularly valuable for maintaining accurate and concise materials databases, which is a prerequisite for high-throughput computational materials discovery.

## Conclusions

In this work, we have proposed a material fingerprinting method based on electronic band structures and demonstrated its advantages over structure-based methods, complementing existing techniques employed for material similarity search. We applied our fingerprinting and clustering framework (elf) to two-dimensional materials exhibiting flat bands to determine chemical and electronic property trends, elucidating multiple chemical and structure groups for further investigation. This fully unsupervised approach is a stepping stone in the realisation of the autonomous materials discovery paradigm.

Similarity search in material properties has become one of the main challenges in modern materials science. Very recently, Google Deepmind significantly increased the number of known stable crystals with GNoME (Graph Neural Networks for Materials Exploration)^1^, releasing an unprecedented number of candidate materials. In this work, we have demonstrated for the first time that material fingerprints deep-learned from electronic band-structure features prove to be a robust tool in linking computationally generated materials to already synthesised compounds exhibiting important emergent properties. This will help widen the bottleneck between material prediction and synthesis of the most promising candidate materials, a crucial task as we move into the new paradigm of AI-driven materials discovery.

In future work, we plan to apply our approach to analyse larger databases, such as the Materials Project, containing 3D materials, and investigate different electronic phenomena emerging from non-trivial band features such as high *T*_*c*_ superconductivity and novel topological phases. Additionally, optimising the material clustering pipeline will be essential, with a focus on methods that prioritise the underlying physics of the materials. One possible approach could involve using dimensionality reduction algorithms to establish broader material groups before applying clustering algorithms in the full fingerprint space. This approach could itself be automated with machine learning, provided key physical properties remain central.

## Methods

### Network training and fingerprint

Previous studies have employed simple fingerprint vectors directly extracted from the electronic band structure of a material^48,49^ to cluster materials’ electronic properties. However, when applying similar techniques to the subset of flat-band materials from 2Dmatpedia, we found that the results were dominated by noise, with materials relatively uniformly spread across fingerprint space. This issue mainly arises from the reliance of those techniques on integrated variables of all electronic bands in some energy range, like the density of states (DoS). As a result, materials sharing meaningful electronic properties may be far apart in fingerprint space if they happen to have a different number of bands passing through some energy range.

To address this issue, we have proposed a fingerprint, based solely on the electronic band structure features of a material. The autoencoder we trained to encode the band feature fingerprints is based on the ResNet series of convolutional neural networks^50,51^, which have found extensive applications in feature extraction problems across the medical and physical sciences^52,53^. We used the first 16 layers of ResNet18 as an encoder and ‘transposed’ it by replacing convolutions with deconvolutions to obtain the decoder. The resulting network is fully convolutional and features skip connections and batch normalization layers, which are characteristic of ResNet models and enable the deep model to converge. We also tested ResNet34 and ResNet50 as backbones for elf, but did not observe a significant improvement; see Supplementary Note 2 for details.

To train the network, we plotted band structure data within a  ± 4 eV range of the Fermi energy, binarized the plots, and resized them as 224 × 224 pixel images. Limiting the energy range to this region around the Fermi energy focuses on the crucial features of the band structure, preventing excessive uniqueness in materials that could lead to reduced clustering power.

The input to the network is a 224 × 224 matrix of zeros and ones, representing the pixels of the band structure image. The network takes 3 layers of size 224 × 224 to represent the RGB colour values of the pixels but these are redundant for our black and white images. The network then predicts an output matrix of the same size as the input. When this output matrix is plotted, we obtain a prediction of the input band structure, based only on the information from the compressed latent layer representation in the centre of the network.

During the optimization process, the network is trained to minimise the binary cross-entropy (BCE) loss^54^ between input and output images. To generate an accurate prediction of the input image from the much smaller set of numbers in the latent representation, the network is forced to encode compressed features of the band structure image in this latent space. Additionally, during training, we applied random noise to regions of the input images with a probability of 0.5.

To achieve a balance between the flexibility needed for high reconstruction accuracy and the dimensionality reduction required for improved clustering, we chose a flattened length of 98 for the latent space. The latent space size was set to a 7 × 7 matrix with two parallel channels. With this network architecture and the application of noise during training, we observed the training loss stabilise at 0.282 (using BCE) after 30 epochs. Performance of the network on the validation set remained within 5% of the training loss throughout the training process.

We can interpret the physical features learned by the network by inspecting its latent space representations. Due to the purely convolutional architecture of the network, we expect soft correlation between specific regions of the input image and specific regions of the latent space matrix. To visualise this, we run the band structure of 2dm-1’s (IrF_2_) through the encoder, and systematically varied the value of one dimension of the resulting encoded representation by a Δ. The full set of slightly altered latent space values is then decoded, and any effect of the change will be observed in the features of the reconstructed image.

**Fig. 6:**
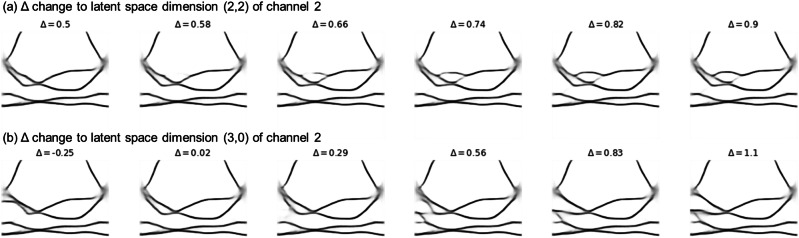
Decoded band structures showing the effect of changing one dimension of 2dm-1’s latent space. Panels **a** and **b** correspond to Δ changes in the latent dimensions [2,2] and [3,0] respectively, of channel 2. The ranges of Δ have been chosen to display the alteration of a single feature in the band structure. With larger ∣Δ∣ values, different possible band crossings and splittings can emerge in the same region.

We changed the latent dimension of channel 2 at matrix position (2,2) by a value Δ in the range 0.5 to 0.9, and the dimension at matrix position (3,0) was changed by Δ in the range of 0.25 to 1.1. The resulting reconstructions are displayed in Fig. 6, with Δ = 0 corresponding to the material’s original band structure.

We observe that, because of the learned features, the auto-encoder can generalise to generate entirely sensible band structures of materials that, in theory, do not exist, by simple manipulation of the latent space. This helps to elucidate the meanings of the individual latent space dimensions. Moreover, due to the compression, there is generally overlap in the latent space regions, and this overlap can help obtain band structures of two seemingly different material groups by continuously tuning some parameters of the latent vector. However, that exercise is out of the scope of this article.

### Multi-stage clustering module

To classify the machine-learned fingerprints, we employed a completely unsupervised multi-stage algorithm. HDBSCAN^29^ was first used to discern regions of high density in the fingerprint space and suggest a hierarchical structure for these clusters. This serves as a stringent identifier of band structure feature similarity among the materials. HDBSCAN, being density-based, facilitates much more general cluster shapes compared to the common ellipsoid-based k-means method. Additionally, it allows us to obtain hierarchical cluster information without being as sensitive to noise in the data (from structural distortions).

To offer a complementary and independent view of our 98-dimensional fingerprint space, the Uniform Manifold Approximation and Projection (UMAP) algorithm was used. UMAP excels at dimensional reduction while preserving the local and global distance relations of points^30^. This sets it apart from other approaches such as Locally Linear Embedding (LLE)^55^ and Hessian Eigenmaps^56^. Furthermore, to visualize relations between different clusters, we used another clustering technique DBSCAN, which allows even arbitrary-shaped connections to create larger groups.

The t-SNE algorithm^57^ provides an alternative to UMAP as it also preserves both the local and global structure of the feature space^58^. In our analysis, we did not observe significant improvement in the embedding space when using t-SNE compared to UMAP. However, UMAP has been extensively applied in bioinformatics research, showing results broadly consistent with t-SNE while offering superior run times and better preservation of global structures^59,60^, such as distances between cell types. Hence, the UMAP algorithm was chosen for our analysis while the comparative embedding results from t-SNE can be found in the Supplementary Note 1.

The Minkowski distance with exponent *p* = 0.2 was used during the clustering process, as this metric is known to scale better than Euclidean (*p* = 2) and Manhattan (*p* = 1) metrics to high dimensional vector spaces^61^.

The two primary free parameters of HDBSCAN (minimum cluster size, *N*_*c*_, and minimum sample size, *N*_*s*_), were optimised by considering their effect on three metrics quantifying the quality of the resulting clustering solution. These were the number of clusters formed, the number of unclassified materials, and the ‘density based clustering validation’ (DBCV) index^62^. The DBCV index evaluates the compactness of a clustering solution by comparing the sparseness of clusters (based on the point in the cluster with the largest core distance measure) with the inter-cluster separation. Thus, a higher value indicates more compact, well-separated clusters and an overall better clustering solution.

**Fig. 7:**
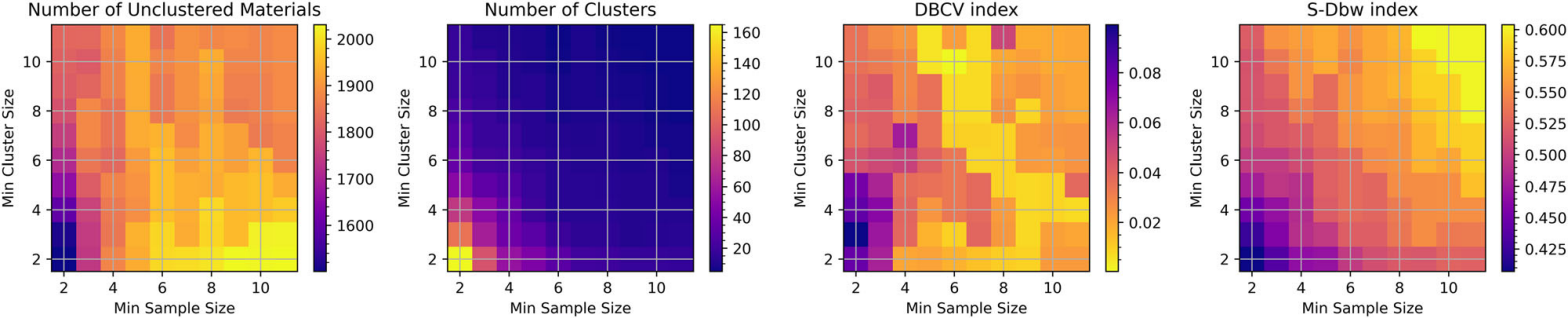
Four metrics for assessing validity of the clustering solutions. The metrics are plotted as function of HDBSCAN's minimum cluster size and minimum sample size variables. The number of clusters formed and the number of unclustered materials together indicate how fine-grained the similarities are between all the materials in a given cluster. The DBCV and S-Dbw indices directly quantify the quality of a given clustering solution with a higher score indicating a better solution with clusters that are less diffuse and more separated from each other.

*N*_*s*_ and *N*_*c*_ were both varied from 2 to 11, and the metrics above were calculated for the resulting clustering solution. These are displayed as colour maps in Fig. 7. Initially, the number of unclassified materials increases, indicating the presence of many difficult-to-classify materials that get forced out of clusters as the clustering parameters become more stringent, requiring larger and more compact clusters. These behaviours are typical of material clustering solutions^11^ using this approach. Considering these factors, we chose *N*_*c*_ = 5 and *N*_*s*_ = 2. This achieves a relatively large DBCV index while minimising the number of unclassified materials and keeping the number of clusters bounded enough to effectively represent the major band structure groups among flat-band materials.

For UMAP, a Nearest Neighbour (NN) parameter of 10 and Minimum Distance (MD) of 0.1 were found to give the optimal 2D representation and general agreement with the HDBSCAN clusters. Finally, for DBSCAN, the parameter *ϵ* = 25 was chosen which allowed identifying visibly separate regions in the UMAP projection. UMAP was mainly a visualisation tool in this work. For clustering, we used HDBSCAN, which prevents the output from being highly sensitive to dimensionality reduction parameters, such as the UMAP Nearest Neighbour parameter, for which it is difficult to predict the precise effect that changes will have on the shape of the embedding space.

## Supplementary information

Supplementary Materials

## Data Availability

All relevant data are available at our HuggingFace repository: Elf_encoded_flat_band_materials.

## Code availability

The code used to generate the results discussed in this work is available from our public Huggingface repository: Elf_encoder.
