# Supplementary material for: Elf autoencoder for unsupervised exploration of flat-band materials using electronic band structure fingerprints
Source: Commun Phys. 2025 Jan 17;8(1):25. doi: 10.1038/s42005-025-01936-2 (PMC11756449; doi:10.1038/s42005-025-01936-2)
Supplement: Supplementary file 1 — Supplementary Materials [file 42005_2025_1936_MOESM1_ESM.pdf]

Supplementary Information for: Elf autoencoder for unsupervised exploration of flat-band materials using electronic band structure fingerprints

Henry Kelbrick Pentz, Thomas Warford, Ivan Timokhin, Hongpeng Zhou, Qian Yang, Anupam Bhattacharya, Artem Mishchenko

Supplementary Note 1: UMAP vs t-SNE

When using t-SNE on the same fingerprint space from our analysis, the major difference is less exaggeration of the same separations that are also present in the UMAP embedding. Overall, by comparing t-SNE in Supplentary Fig. 1b to UMAP in Supplementary Fig. 1a, very similar global structure is found in both embedding plots. For example, the cluster groups (6,22,0,18 etc), (26,27,31-33) and (5,13-15,17) forming a loop that breaks off from the arrow-shaped main island which contains the majority of the unclustered materials. With UMAP, some distances are exaggerated which exemplifies some features like the separation of clusters (26,27,31-33) from the main island while also compressing some relationships like those between the clusters of the main island (e.g. 38-40 and 45-49). Ultimately the global relationships represented in our data are similar when both algorithms have been initialised in an appropriate deterministic manner with PCA. However, ultimately UMAP was chosen for its superior run times and the fact it has been shown, in general, to better preserve global structures in data [1][2][3].

Supplementary Note 2: ResNet18 model

Many state-of-the-art (SOTA) methods for image clustering continue to be based on the convolutional ResNet models. The current benchmark leaderboard for image clustering on the CIFAR-10 dataset can be found at https://paperswithcode.com/sota/image-clustering-on-cifar-10, with five of the top ten approaches having a ResNet18 backbone for their image encoder (and two with ResNet34), as of august 2024. Some advances have been made with transformer based image encoder models (A. Radford et al., 2021, https://arxiv.org/abs/2103.00020) but in general the accuracies achieved are comparable to the SOTA approaches that use convolutional encoders.

The primary difference between older approaches and SOTA ones, as opposed to the model backbone, is the use of transfer learning (i.e. pre-training on large datasets). The resulting models excel at general image encoding and clustering tasks. However, because the features and underlying rules of electronic band structures are unlikely to have been well-represented in any general pre-training dataset, it's unlikely that any approach with transfer learning would offer an advantage over a ground-up ResNet18 model, when applied to learning the specific features of electronic band structure images from 2dmatpedia.

The larger models ResNet34 and ResNet50 were also considered for our analysis but no improvements in accuracy over ResNet18 were observed after 30 epochs of training. Further testing, summarised in Supplementary Fig. 2, showed that ResNet34 achieved a small reduction of 0.014 in BCE reconstruction loss on the validation set after 45 epochs of training. However, the improvement was minimal enough that we opted to use the simpler ResNet18 model which was also the most computationally efficient.

Supplementary Note 3: Phylogenetic tree construction

The hierarchical structure of the clusters is determined internally by the HDBSCAN algorithm. Clusters are split up during the algorithm's execution due to the variation of a parameter λ, which can be interpreted as an effective minimum allowed cluster density. Clusters that split from each other at a certain value of λ are recorded as branches formed from the parent(lower λ cluster) for the hierarchical tree. Sets of points smaller than the minimum cluster size parameter, which are forced out of the cluster at some λ value, are not recorded as branches and marked as noise in the hierarchy. This process generates a "condensed tree" that records which clusters were linked together at some smaller value of λ. This tree forms the basis of HDBSCAN's final cluster selection process and is what is plotted in the main text as a phylogenetic tree. Further details on the formation of the condensed tree with its relation to, and differences from, single linkage criteria can be found in the HDBSCAN documentation [4] found at https://hdbscan.readthedocs.io/en/latest/how_hdbscan_works.html.

Supplementary References

[1] Ruizhi Xiang et al. "A comparison for dimensionality reduction methods of single-cell RNA-seq data". In: *Frontiers in genetics* 12 (2021), p. 646936.

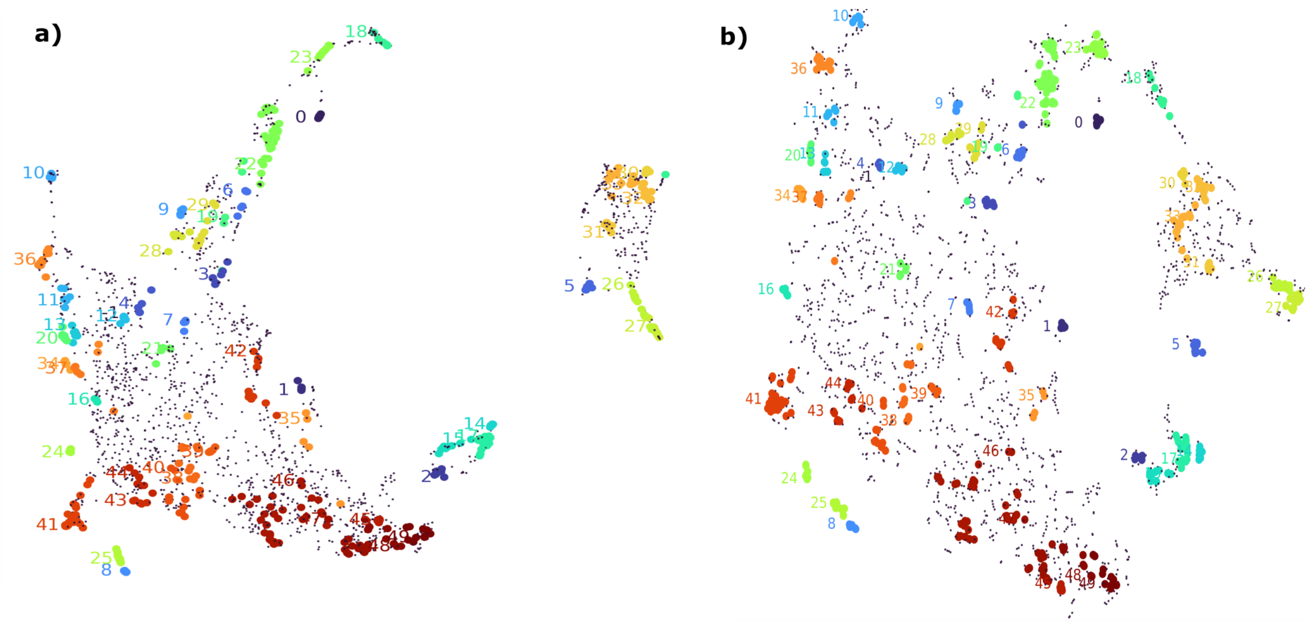

Supplementary Fig. 1: Comparison of UMAP and t-SNE embeddings. **a)** UMAP embedding from the main analysis, included for ease of comparison **b)** t-SNE embedding of the same fingerprint space with perplexity = 10, and early exaggeration = 12.

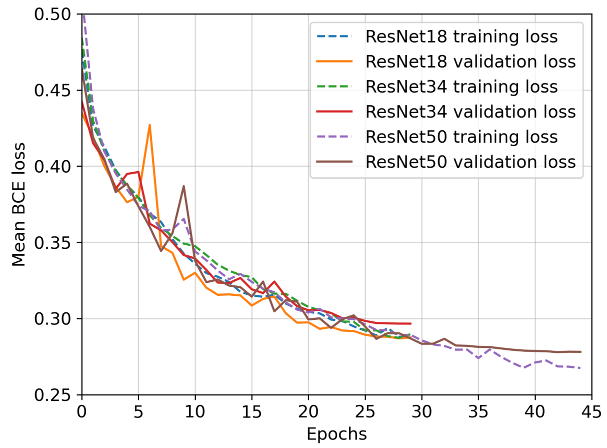

Supplementary Fig. 2: Training performance of different ResNet models. BCE training and validation losses plotted against training epochs for ResNet18 and ResNet34 trained up to 30 epochs and for ResNet50 training up to 45 epochs.

[2] Carlos P Roca et al. “A cross entropy test allows quantitative statistical comparison of t-SNE and UMAP representations”. In: *Cell Reports Methods* 3.1 (2023).

[3] Leland McInnes, John Healy, and James Melville. “Umap: Uniform manifold approximation and projection for dimension reduction”. In: *arXiv: 1802.03426* (2018).

[4] Ricardo J. G. B. Campello, Davoud Moulavi, and Joerg Sander. “Density-Based Clustering Based on Hierarchical Density Estimates”. In: *Advances in Knowledge Discovery and Data Mining*. 2013, pp. 160–172. ISBN: 978-3-642-37456-2.
